# Downstream Process Intensification for AAV Purification by Affinity Chromatography Using Single Pass Tangential Flow Filtration

**DOI:** 10.1002/bit.70090

**Published:** 2025-10-25

**Authors:** Akshay S. Chaubal, Ronny Horax, Christopher J. Yehl, S. Ranil Wickramasinghe, Xianghong Qian, Lu Wang, Andrew L. Zydney

**Affiliations:** ^1^ Department of Chemical Engineering Pennsylvania State University, University Park Pennsylvania; ^2^ Department of Biomedical Engineering University of Arkansas Fayetteville Arkansas; ^3^ Department of Chemical Engineering University of Arkansas Fayetteville Arkansas; ^4^ Gene Therapy Purification Development, Roche Philadelphia Pennsylvania

**Keywords:** Adeno‐associated virus (AAV), affinity chromatography, clarification, continuous processing, process intensification, Single pass tangential flow filtration

## Abstract

To enable adeno‐associated viral vectors (AAV) to achieve their maximum potential, next‐generation manufacturing processes must be developed to make gene therapies more affordable and accessible. This study focused on the design of two different intensified AAV downstream manufacturing processes at bench and pilot scale. Novel clarification methods were studied at bench scale, including the use of BioOptimal™ MF‐SL tangential flow microfilters for continuous removal of cell debris. Membrane adsorbers were used for further clarification, including DNA removal. Single pass tangential flow filtration (SPTFF) was implemented at bench scale by feeding the clarified cell lysate (CCL) into two Pellicon XL50 cassettes with 100 kDa regenerated cellulose membranes. At pilot scale, a multi‐membrane staged SPTFF module was designed to concentrate 10 L of AAV CCL. Both SPTFF systems provided 12X inline volumetric concentration with AAV yield > 99% after an appropriate buffer chase. Host cell protein removal was 48% and 37% for the bench and pilot scale processes, respectively. As an initial proof‐of‐concept, an integrated process was developed at pilot‐scale which linked clarification, SPTFF, and affinity chromatography. The integrated process offered an 81% reduction in total operating time (due to the reduced volume of load material for the affinity column after preconcentration by SPTFF), 36% improvement in affinity resin utilization (due to the higher AAV concentration in the column load), and an estimated 10% reduction in raw material costs. These improvements translated to an 8.5‐fold increase in overall productivity compared to an equivalent batch process, underscoring the potential for SPTFF to intensify large‐scale AAV downstream processing.

## 1 Introduction

Adeno‐associated viral vectors (AAV) are a proven platform for safe and efficient in vivo gene delivery and are now a cornerstone of the rapidly‐expanding gene therapy clinical pipeline. AAV possess broad tropism and low immunogenicity, which has enabled their utility in treating a wide range of genetic disorders. Commercial applications of AAV‐based gene therapies include treatment of inherited retinal diseases (Alsalloum et al. 2024), neuromuscular disorders such as spinal muscular atrophy (Wu et al. 2025), and blood diseases like hemophilia (Joshi et al. 2024). Despite their breakthrough potential, the high costs of AAV‐based therapies strongly restricts patients’ access, with single treatments sometimes costing as much as $3.5 million USD (Kanmeister et al. 2023). These steep costs can be partially attributed to bottlenecks within the AAV manufacturing process such as high facility/consumable costs, low product yield, and long manufacturing times (Kliegman et al. 2024).

Process intensification – broadly defined as enhancing manufacturing productivity without increasing input resources – has gained traction across the industry as a strategy to debottleneck manufacturing (Crowley et al. 2024). AAV are traditionally produced within human embryonic kidney (HEK293) cells, which are lysed to release the viral capsids into the culture medium. This in turn introduces a significant burden of impurities such as cell debris, host cell proteins (HCPs), and host cell DNA into the product stream. The primary removal of these impurities occurs across two initial downstream steps: clarification to remove large cell debris (with some HCP and DNA removal) and affinity chromatography to remove the bulk of the HCPs and remaining DNA (Lorek et al. 2025). These steps are often associated with the highest costs of goods and longest operating times (Thakur et al. 2023); thus, there is strong interest in intensifying these early‐stage purification steps to enhance overall process efficiency.

AAV clarification is typically performed via depth filtration (Chu, Borujeni, et al. 2023). Although effective in reducing lysate turbidity, traditional depth filters can have highly variable AAV recovery (Cherradi et al. 2020) and low capacity due to the high level of impurities (Chinnawar and Marchand 2022). Several groups have instead explored the use of tangential flow filtration (TFF), alternating tangential filtration (ATF), or tangential flow depth filtration (TFDF) (Kozaili and Strauss 2024; Leach et al. 2025; Hao, Horax, Qian, et al. 2025) for initial clarification, either as an independent clarification step or integrated with a perfusion bioreactor to provide continuous upstream harvest (Mostafavi et al. 2025; Tona et al. 2023; Mendes et al. 2022).

The AAV clarified cell lysate (CCL) is then processed through affinity chromatography, which uses selective ligands to bind the AAV capsids while allowing unwanted HCPs and DNA to flow through (Bogdanovic et al. 2025). Productivity of affinity chromatography is limited by low AAV titers, leading to poor resin utilization and long product loading times. In addition, the prolonged exposure of the resin and bound AAV to nutrient‐rich media increases the potential for microbial contamination (Wang et al. 2025).

**Figure 1:**
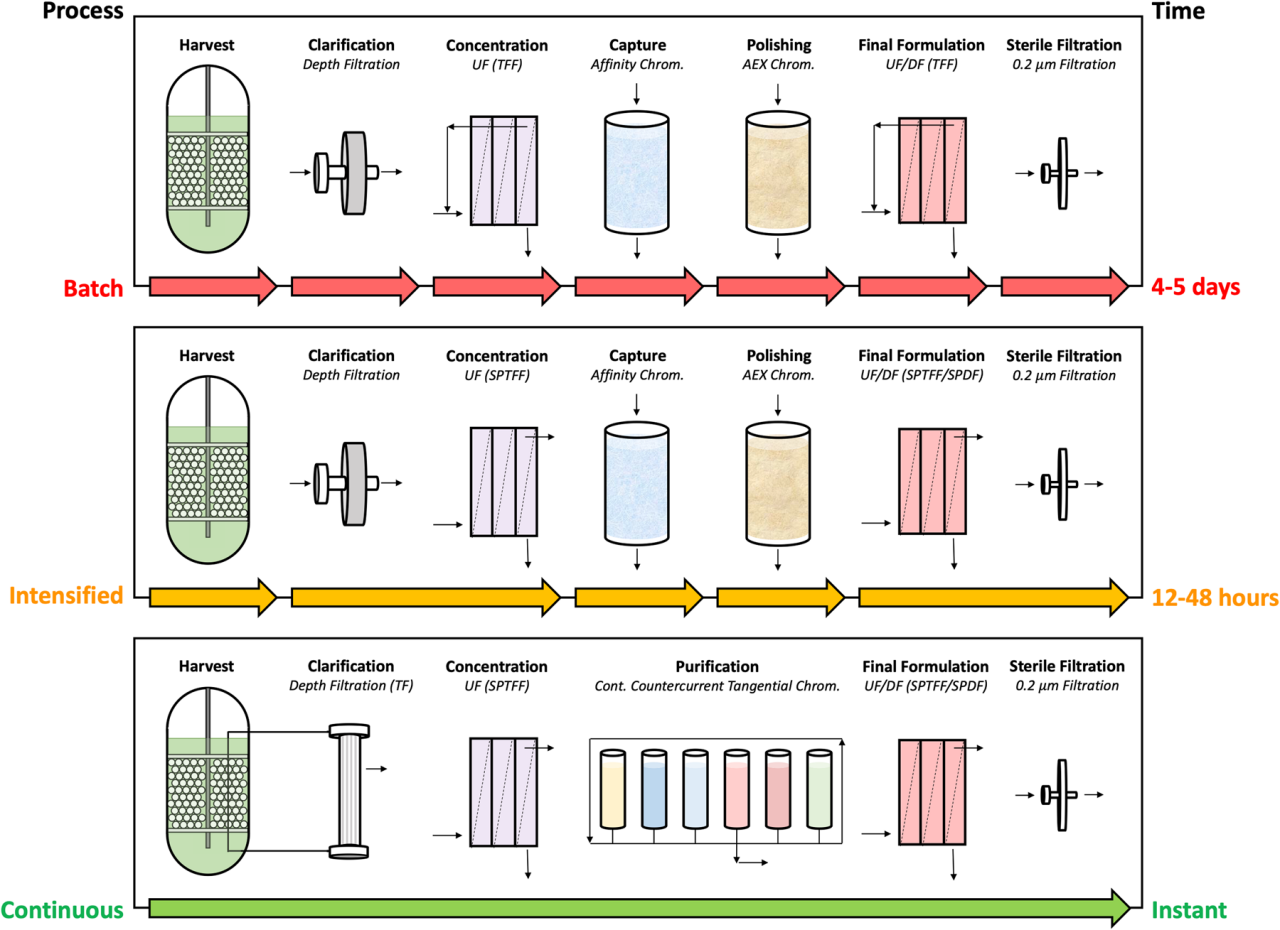
Schematic diagram of AAV downstream manufacturing processes operated in batch (*top panel*), intensified (*middle panel*), and fully continuous (*bottom panel*) modes.

One approach for intensifying the affinity chromatography operation is to preconcentrate the AAV load material using ultrafiltration (UF) (McCarney et al. 2025). However, the repeated cycling of the feed in batch UF can cause AAV aggregation and/or degradation due to the high shear rates in commercial UF modules (Chaubal, Yehl, et al. 2025). A potentially attractive alternative is to employ single pass tangential flow filtration (SPTFF), in which the module is operated at high permeate conversion (defined as the ratio of the permeate to feed flow rate) to achieve significant concentration of retained AAV in a single pass without any recycling (Madsen et al. 2022). SPTFF offers inline volume reduction, which eliminates the need for intermediate storage tanks (Dizon‐Maspat et al. 2012), and the preconcentration of the AAV feed reduces the duration of product loading during capture chromatography (Rahane et al. 2024; Brinkmann and Elouafiq 2021). Previous studies of SPTFF for monoclonal antibody processing has demonstrated that the preconcentration can also increase resin binding capacity (Lutz 2017; Elich et al. 2019). Although the use of SPTFF to intensify AAV affinity chromatography has not been reported in the literature, SPTFF has been employed for continuous concentration/purification of clarified AAV lysate (Chaubal, Yehl, et al. 2025), and it has been proposed for final formulation of viral vectors (Chaubal and Zydney 2023; Heldt et al. 2024, Chaubal, Single, et al. 2025). The successful integration of SPTFF within the AAV downstream process opens opportunities to transition towards intensified and/or fully continuous platforms (Figure 1).

This study focuses on the design of two distinct intensified AAV bioprocesses at bench and pilot scale. AAV clarification at bench scale was performed using BioOptimal™ MF‐SL tangential flow microfilters coupled with anion‐exchange membrane adsorbers to achieve both high AAV yield and high impurity removal. Following clarification, SPTFF was performed using two 50 cm^2^ cassettes connected in series, enabling a 12× volumetric concentration factor (VCF) with nearly 100% AAV yield. The concentrated AAV feed was then processed through affinity chromatography, resulting in an 80% reduction in overall operating time. In contrast, pilot‐scale clarification was designed using conventional Clarisolve™ depth filtration cassettes that are commonly used in industry today. SPTFF at pilot scale was performed using a Cadence™ modular cassette that allows for the use of staged membrane configurations appropriate for processing larger feed volumes (Madsen et al. 2022). The SPTFF retentate was further processed through affinity chromatography, giving an 8.5× improvement in overall productivity compared to conventional batch processes.

## 2 Materials and Methods

### 2.1 AAV Production

Bench‐scale experiments were performed using AAV2 lysate that was produced within suspension cell culture at the University of Arkansas (Fayetteville, AR) as discussed previously (Hao, Horax, Qian, et al. 2025). Briefly, HEK293 cells were grown in Gibco® Viral Vector HEK Medium supplemented with 2% (v/v) GlutaMAX™ (*ThermoFisher Scientific*). The cultures were seeded at an initial density of 0.3 × 10^6^ cells/mL and maintained in a CO_2_ incubator at 37°C and 82% relative humidity. Once the cell density reached ~5.5 × 10^6^ cells/mL, the cell culture was diluted with fresh medium to achieve a final density of 3.0 × 10^6^ cells/mL. The HEK293 cells were then transfected with AAV plasmids, and the culture was incubated for 72 h to allow enough time for AAV production. The cells were lysed by addition of 10% (v/v) AAV‐MAX™ lysis buffer (*ThermoFisher Scientific*) and incubated for 4 h before harvesting.

Pilot‐scale studies were performed with AAV clarified cell lysate (CCL) generated at Spark Therapeutics Inc. (Philadelphia, PA), now part of Roche. Proprietary recombinant AAV capsids were produced in HEK293 cells via the triple transfection method. Following AAV production, the host cells were lysed at 37°C using 0.1% (v/v) Triton X‐100, 2 mM MgCl_2_, and 50 units/mL of benzonase to generate the feed material for pilot‐scale experimentation.

### 2.2 Clarification

AAV2 clarification at bench scale was performed using BioOptimal™ MF‐SL tangential flow microfilters provided by Asahi‐Kasei Bioprocess (Glenview, IL) as shown previously (Hao, Wickramasinghe, et al. 2025, Hao, Horax, Qian, et al. 2025). The feed flow rate was set at 160 mL/min using a Masterflex® peristaltic pump to maintain a wall shear rate of 2000 s^−1^, while the permeate flow rate was set at 1.66 mL/min, corresponding to a permeate flux of 20.0 L/m^2^/h (LMH), using a second pump placed on the permeate outlet line. Membrane fouling was quantified by monitoring the system TMP using pressure transducers purchased from Repligen Corporation (Waltham, MA):

(1) $$\mathrm{TMP}=\frac{P_{f}+P_{r}}{2}-P_{p}$$

where P_f_, P_r_, and P_p_ correspond to the pressure at the feed inlet, retentate outlet, and permeate outlet ports, respectively.

Secondary clarification was performed using 3M™ Polisher ST capsules purchased from Solventum Co. (Minneapolis, MN). The 3M™ Polisher ST is an anion‐exchange (AEX) adsorptive filter containing membranes functionalized with quaternary ammonium and guanidinium groups that preferentially bind negatively‐charged impurities (Hester et al. 2020). 1 and 25 cm^2^ 3M™ Polisher ST membrane capsules were operated at a constant flux of either 240 or 600 LMH. The flux was set using a Masterflex® pump placed upstream of the module, with the TMP measured using a SciLog® pressure sensor placed at the system inlet.

AAV clarification at pilot‐scale was performed using an industry‐standard depth filtration process, which consisted of appropriately‐sized Clarisolve™ MS20 depth filters connected in series with a 0.2 μm pore‐sized bioburden reduction filter. The flux through the depth filter was maintained at 150 LMH.

### 2.3 Single Pass Tangential Flow Filtration (SPTFF)

SPTFF was performed at bench scale using two Pellicon® XL50 cassettes connected in series. Each cassette was fitted with 100 kDa Ultracel® membranes with an area of 50 cm^2^. The cassettes were mounted in a vertical orientation to facilitate removal of entrapped air, with the retentate port on the first cassette directly connected to the feed port on the second cassette giving a total filtration area of 100 cm^2^. The permeate streams exiting both cassettes were left open to the atmosphere and were collected independently. The system TMP was monitored using three Repligen pressure transducers placed at the feed inlet port of the first cassette, the retentate outlet port of the second cassette, and the permeate outlet from the first cassette. The volumetric concentration factor (VCF) was controlled using two Masterflex® peristaltic pumps, one on the feed inlet and one on the retentate exit. The VCF is defined as the ratio of the feed flow rate (Q_f_) to the outlet retentate flow rate (Q_r_):

(2) $$\mathrm{VCF}=\frac{Q_{f}}{\mathrm{Qr}}$$

The overall retentate and permeate flow rates were evaluated by timed collection using digital scales to collect the outflow from the SPTFF system.

At pilot scale, SPTFF was performed using fifteen Centramate® T02 cassettes with 100 kDa PES membranes (*Cytiva Life Sciences*), providing a total membrane area of 0.279 m^2^. The modular capabilities of the Centramate® membranes allowed for customizable cassette staging during large‐scale SPTFF runs; an 8‐stage module was constructed with a configuration of 3‐3‐2‐2‐2‐1‐1‐1 as described subsequently. The system was operated with pumps, scales, and pressure transducers as described earlier. SPTFF experiments were always preceded by appropriate flushing with water and equilibration with buffer (20 mM Tris, 300 mM NaCl buffer, pH 8.0) as recommended by the manufacturer.

### 2.4 Affinity Chromatography

Affinity chromatography at bench scale was performed using POROS™ GoPure™ AAVX pre‐packed columns (*ThermoFisher Scientific*) connected to an ÄKTA™ Pure chromatography system. Product loading was performed at a flow rate of 0.33 mL/min, corresponding to a 3 min residence time, as suggested by the manufacturer. Following loading, the column was washed with 20 mM Tris, 150 mM NaCl, and 0.01% Pluronic F‐68 at pH 8.0 to remove residual unbound impurities. The bound AAVs were eluted at a flow rate of 0.6 mL/min using 100 mM glycine‐HCl + 0.01% Pluronic F‐68 at pH 2.5. The eluate was collected as 1 mL fractions into conical tubes containing 0.2 mL of neutralization buffer (1 M Tris‐HCl at pH 8.4). Following elution, the resin was regenerated by flushing 0.2 M phosphoric acid for 15 min, followed by 6 M guanidine‐HCl for 15 min as per the manufacturer's instructions. All bench scale runs were performed using the same affinity column.

Pilot scale chromatography was performed using AVB Sepharose High Performance (HP) resin (*Cytiva Life Sciences*) packed into Omnifit® EZ columns (*Diba Industries*). The different affinity resins for the bench and pilot scale processes reflects the different AAV serotypes; screening data indicated that the AVB resin provided better performance with the proprietary AAV produced at Spark/Roche. Data obtained at 10 L used an ÄKTA™ Pure chromatography system, while data from a 500 L run used an ÄKTA™ Pilot system. The residence time during product loading was again set to 3 min, with the subsequent wash, elution, and neutralization steps following a similar protocol as described above. The AAV was eluted at pH 3.0 into a vessel containing neutralization buffer at pH 8.5.

### 2.5 Assays

Quantitative polymerase chain reaction (qPCR) was used to evaluate the AAV titer. DNA content was measured using standard Quant‐iT™ PicoGreen™ dsDNA assay kits purchased from ThermoFisher Scientific. For bench scale experiments, HCP content was quantified using a colorimetric Bradford protein assay. Although the Bradford assay is unable to differentiate between AAV capsid proteins and host cell protein impurities, the contribution of AAV capsids to the overall protein concentration within the clarified lysate was < 1%. Therefore, the signal from the Bradford assay provides an accurate estimate of the HCP concentration. At pilot scale, HEK293 HCP ELISA Kits (*Cygnus Technologies*) were used to quantify HCPs without interference from the AAV. Further details behind the operating procedure(s) for these assays can be found in the literature (Hao, Horax, Qian, et al. 2025; Hao, Wickramasinghe, et al. 2025, Hao, Horax, Wickramasinghe, et al. 2025).

## 3 Results

### 3.1 Biooptimal™ MF‐SL Clarification at Bench Scale

**Figure 2:**
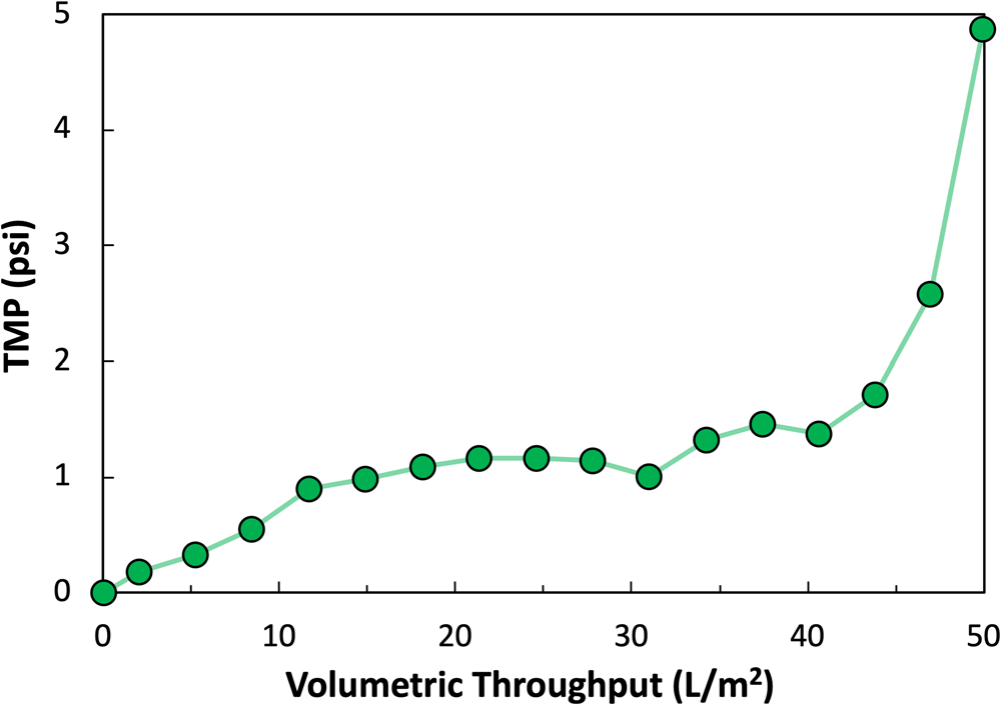
TMP vs volumetric throughput during AAV2 clarification through a 0.005 m^2^ BioOptimal^TM^ MF‐SL filter. Data collected with a 300 mL feed batch at a constant flux of 15.6 L/m^2^/h.

Bench‐scale clarification was performed using new or regenerated 50 cm^2^ BioOptimal™ MF‐SL hollow fiber modules operated in tangential flow mode. AAV2 lysate was fed into the system at a feed flow rate of 160 mL/min, with the permeate flow rate set at 1.66 mL/min (corresponding to an inlet wall shear rate of 2000 s^−1^ and a permeate flux of 20 L/m^2^/hr). Experiments were conducted with approximately 300 mL of AAV2 harvest material, with the system TMP plotted as a function of volumetric throughput in Figure 2. The TMP remained relatively stable over the initial phase of the clarification but increased rapidly when around 80% of the feed material had been processed due to the high concentration of retained cell debris at the end of the run. The BioOptimal™ MF‐SL membrane filter reached 40 L/m^2^ throughput with the TMP remaining well below the maximum recommended value of 21.8 psi throughout the runs.

AAV yield and impurity removal through the BioOptimal™ MF‐SL filter for the ~300 mL harvest batch is presented in Table 1. AAV transmission through the membrane (defined as the measured AAV concentration in the permeate divided by that in the initial feed) was 100 ± 2%, where the 95% confidence interval is determined from the triplicate measurements. The total AAV yield was 93%, with the small loss arising primarily from the hold‐up volume within the retentate channel/tubing. This loss could likely be significantly reduced with a post‐use buffer flush, although that would cause some dilution of the AAV product. Although the BioOptimal™ MF‐SL filter provided high removal of cells and cell debris, there was significant transmission of host cell proteins (HCPs) and host cell DNA, consistent with the relatively large nominal pore size (0.4 µm). The feed contained 1.80 ± 0.08 mg/mL of total HCP; this high HCP content is due to the release of HCP upon lysis of the HEK293 cells to release the AAV (Chu, Shastry, et al. 2023). The clarified permeate contained 1.65 mg/mL of HCP and 20 μg/mL of host cell DNA, corresponding to 8% and 21% reductions, respectively. In a separate biological replicate run with a BioOptimal™ MF‐SL module maintained at the same operating conditions (summarized in Table S1 in the Supplementary Information), the AAV yield was 90%, with reductions in HCP and DNA concentrations of 18% and 29%, respectively. During AAV production at a laboratory scale of 1000 mL or less, there are slight batch‐to‐batch variations in AAV titer, turbidity, and impurity content leading to some variability in filter performance.

**Table 1:** Concentration of AAV, HCP, and DNA within feed, permeate, and retentate streams following BioOptimal^TM^ MF‐SL clarification of a 289 mL AAV2 harvest batch.

Sample	Volume (mL)	Turbidity (NTU)	AAV (vg/mL)	AAV fraction of feed (%)	HCP (mg/mL)	DNA (μg/mL)
Feed	289	552	3.12 ± 0.17 × 10^11^	N/A	1.80 ± 0.08	25.3 ± 0.6
Permeate	269	57	3.13 ± 0.46 × 10^11^	93 ± 14%	1.65 ± 0.04	20.0 ± 0.4
Retentate	20	***	4.13 ± 0.02 × 10^11^	9 ± 1%	2.28 ± 0.08	52.8 ± 0.6

*** Retentate turbidity was very high due to high concentration of retained cell debris.

### 3.2 Impurity Reduction Using Adsorptive Filters

**Figure 3:**
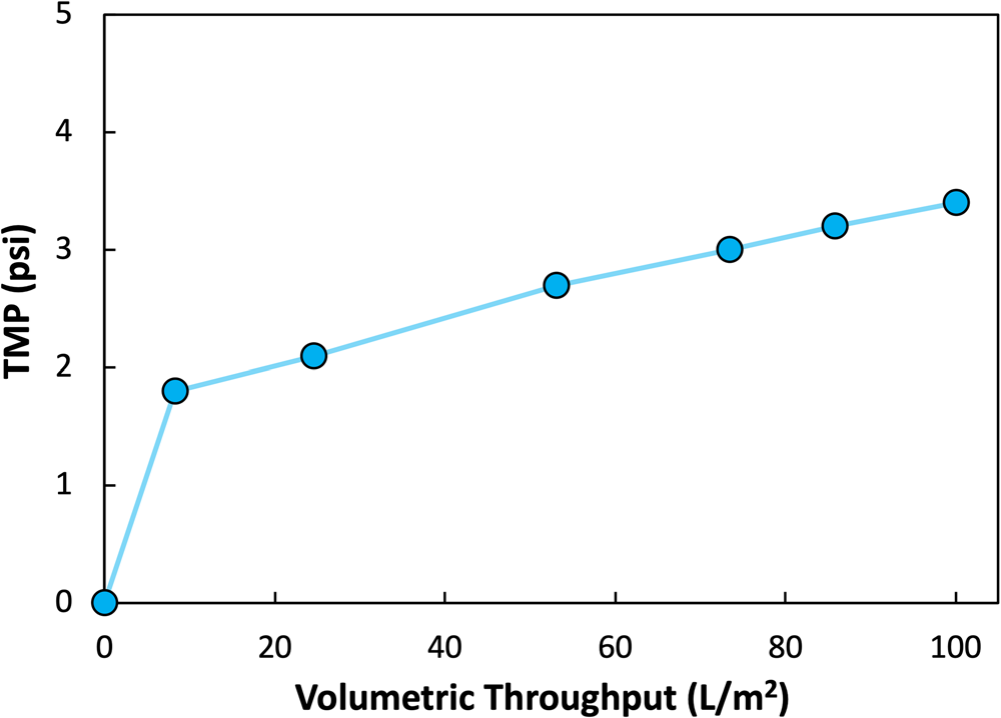
TMP vs volumetric throughput during filtration of 200 mL of the permeate from the BioOptimal^TM^ MF‐SL through a 25 cm^2^ 3M^TM^ Polisher ST capsule at a constant feed flux of 240 LMH.

To further reduce the concentration of host cell impurities, the permeate from the BioOptimal™ MF‐SL filter was processed through a 3M™ Polisher ST capsule containing cation‐functionalized membranes that target the removal of negatively charged impurities. Approximately 200 mL of the BioOptimal™ MF‐SL permeate was filtered through a 25 cm^2^ Polisher capsule, giving an AAV mass loading of 2.46 × 10^16^ viral genomes (vg) per m^2^ of membrane, at a feed flow rate of 10 mL/min (feed flux of 240 LMH). The TMP remained well below the maximum recommended pressure differential of 35 psi (data in Figure 3). The final turbidity of filtrate collected from the 3M™ Polisher ST typically fell below 35 NTU.

The performance of the Polisher ST membrane across three separate runs is summarized in Table 2. The 25 cm^2^ Polisher reduced the HCP level by 88% with > 99% DNA removal. However, AAV yield was only 53%, which suggests that a significant amount of AAV capsids were bound to the membrane. Additional experiments were performed using smaller Polisher devices (with 1 cm^2^ area, either alone or two in parallel) to examine the effect of flux and loading on Polisher performance. The AAV yield increased with increasing mass loading, approaching 100% at a loading of 1.45 × 10^17^ vg/m^2^. However, the DNA and HCP removal were reduced at high mass loading, suggesting that the binding sites within the Polisher capsule become completely saturated at a sufficiently high mass loading. Additionally, there is some evidence that DNA removal may also decrease with increasing filtrate flux (note the large reduction in DNA removal compared to that for HCP in the first two runs). This may reflect some type of mass transfer limitation on DNA binding to the Polisher ST membrane(s).

**Table 2:** Yield of AAV and removal of HCP and DNA as a function of AAV mass loading using the 3M^TM^ Polisher ST.

Flux (LMH)	Membrane area (cm^2^)	AAV Mass loading (vg/m^2^)	AAV yield	HCP removal	DNA removal
240	25	2.46 × 10^16^	53 ± 2%	88 ± 4%	> 99 ± 1%
600	1	3.38 × 10^16^	65 ± 14%	80 ± 4%	88 ± 2%
600	2 × 1	1.45 × 10^17^	100 ± 11%	50 ± 2%	87 ± 3%

### 3.3 SPTFF at Bench Scale

Following AAV2 clarification through the BioOptimal™ MF‐SL and 3M™ Polisher ST, the clarified material was split into two experimental arms: 110 mL of material was saved for direct loading to the affinity column (control arm), while 148 mL was preconcentrated via SPTFF before the affinity chromatography. The SPTFF process used two Pellicon XL 50 cm^2^ cassettes connected in series with 100 kDa regenerated cellulose (RC) membranes. The SPTFF feed had 1.2 × 10^11^ vg/mL of AAV, 0.2 mg/mL HCP, and less than 0.01 μg/mL DNA; the very low DNA concentration is due to the high degree of DNA removal by the Polisher ST. The SPTFF system was operated at a feed flow rate of 1.00 mL/min (feed flux = 6 L/m^2^/h) and a retentate flow rate of 0.082 mL/min corresponding to ~92% conversion or VCF = 12. While it is technically feasible to push SPTFF to higher concentration factors, doing so may result in AAV aggregation. SPTFF operation under the tested condition was stable, with minimal increase in TMP over the entire 15 L/m^2^ batch and no evidence of AAV aggregation. Linear extrapolation of the gradient in TMP suggests that the capacity of the SPTFF step would be above 1000 L/m^2^ (see Figure S1 in the Supplementary Information).

The concentration of AAV in the collected retentate was 1.24 × 10^12^ vg/mL, which equates to an ~11× concentration factor or an 89% AAV yield. Analysis of permeate samples collected during SPTFF showed that the 100 kDa RC membranes were fully retentive to the AAV capsids (< 2% AAV transmission), and there was no evidence of any AAV degradation. Instead, the “lost” AAV were likely accumulated within a concentration polarization boundary layer that forms above the membrane surface (Chaubal and Zydney 2023). The AAV yield can be increased to nearly 100% by flushing clean buffer through the retentate channels of the two cassettes, without ultrafiltration (i.e., with the permeate ports closed). The buffer chase also recovered some HCPs, with the overall HCP removal decreasing slightly from 54% to 48% (Table 3). Furthermore, the 8.3 mL of buffer chase diluted the final AAV to a concentration of 8.68 × 10^11^ vg/mL giving an overall concentration factor of 7.3. An additional biological replicate run performed with a different batch of AAV CCL confirmed that SPTFF consistently provides AAV yield > 96% under similar conditions, thereby demonstrating process robustness (data provided in Table S2 in Supplementary Information).

**Table 3:** Performance of bench scale SPTFF performed with two Pellicon XL 50 cm^2^ cassettes in series using 100 kDa regenerated cellulose membranes. Results are shown before and after an 8.27 mL buffer chase performed at the end of the run.

	Volume reduction factor	AAV concentration factor	AAV yield	HCP removal
**Before buffer chase**	11.8	10.5	89 ± 3%	54 ± 2%
**After buffer chase**	7.3	7.3	103 ± 3%	48 ± 2%

### 3.4 Affinity Chromatography at Bench Scale

The clarified AAV material was then processed through affinity chromatography to determine the effect of the SPTFF preconcentration on AAV yield and process productivity. Specifically, 110 mL of unconcentrated material and 17 mL of the 7.3× SPTFF‐preconcentrated material were loaded onto a 1 mL pre‐packaged POROS™ GoPure™ AAVX affinity column (*ThermoFisher Scientific*). The same column was used for both runs, with appropriate resin regeneration carried out between runs per the manufacturer's instructions. In both cases, material was loaded at a flow rate of 0.33 mL/min to achieve a 3‐min residence time. Elution was performed at pH 2.5, with the eluate peak manually fractionated into 1.2 mL increments to assess overall AAV yield for both experimental arms.

**Figure 4:**
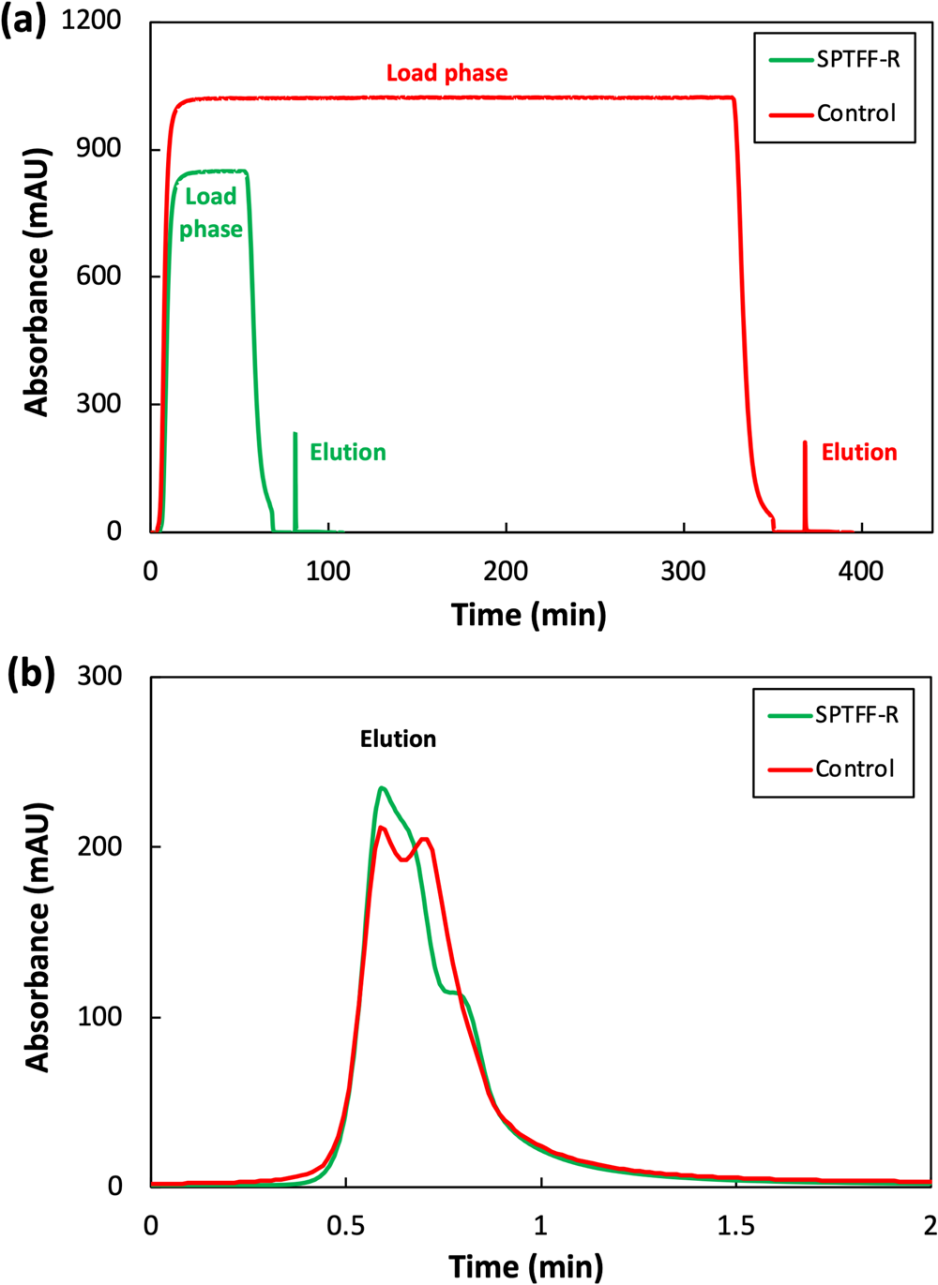
(a) UV absorbance at 280 nm during affinity chromatography with 110 mL of unconcentrated control material (in red) and 17 mL of 7.3× SPTFF‐preconcentrated material (in green). (b) Overlaid elution profiles of unconcentrated control material (in red) and SPTFF‐preconcentrated material (in green). “*t* = 0” refers to the start of the elution step.

The full affinity chromatograms are presented in Figure 4a, with UV absorbance at 280 nm plotted as a function of operating time. A major difference between the two runs is the duration of the product loading phase (flat plateau region of the chromatogram) – only 70 min were required to load the preconcentrated material, compared to 350 min for the unconcentrated material. The reduced loading time for the preconcentrated feed addresses two aspects of AAV degradation: minimizing the time AAV binds to the resin, where capsid proteins can fold unnaturally (Khanal et al. 2023; Guapo et al. 2023), and reducing the prolonged presence within the enriched CCL matrix, which contains small‐sized enzymes that may cause AAV degradation.

Figure 4b shows an enlarged view of the elution peaks, with *t* = 0 set as the start of the elution. The peak areas are nearly identical for the two runs, demonstrating that preconcentration by SPTFF had no adverse effect on AAV recovery – both runs achieved AAV yield > 96%. However, analysis of flowthrough samples obtained during the binding step revealed that the preconcentrated material contained 83% less AAV in the flowthrough. This suggests that the SPTFF preconcentration improves resin binding capacity, potentially due to the increased AAV titer (greater driving force for binding) and lower concentration of impurities (reduced nonspecific binding). Since the AAV yield in these experiments was quite high, this reduction in AAV concentration in the flowthrough had minimal effect on the overall process yield. However, some AAV serotypes show much poorer binding during affinity chromatography, in which case the preconcentration by SPTFF can lead to significant improvements in yield (as discussed subsequently). These findings highlight the potential of using SPTFF to boost process productivity via reductions in operating time and improvements in AAV yield.

### 3.5 SPTFF at Pilot Scale

**Figure 5:**
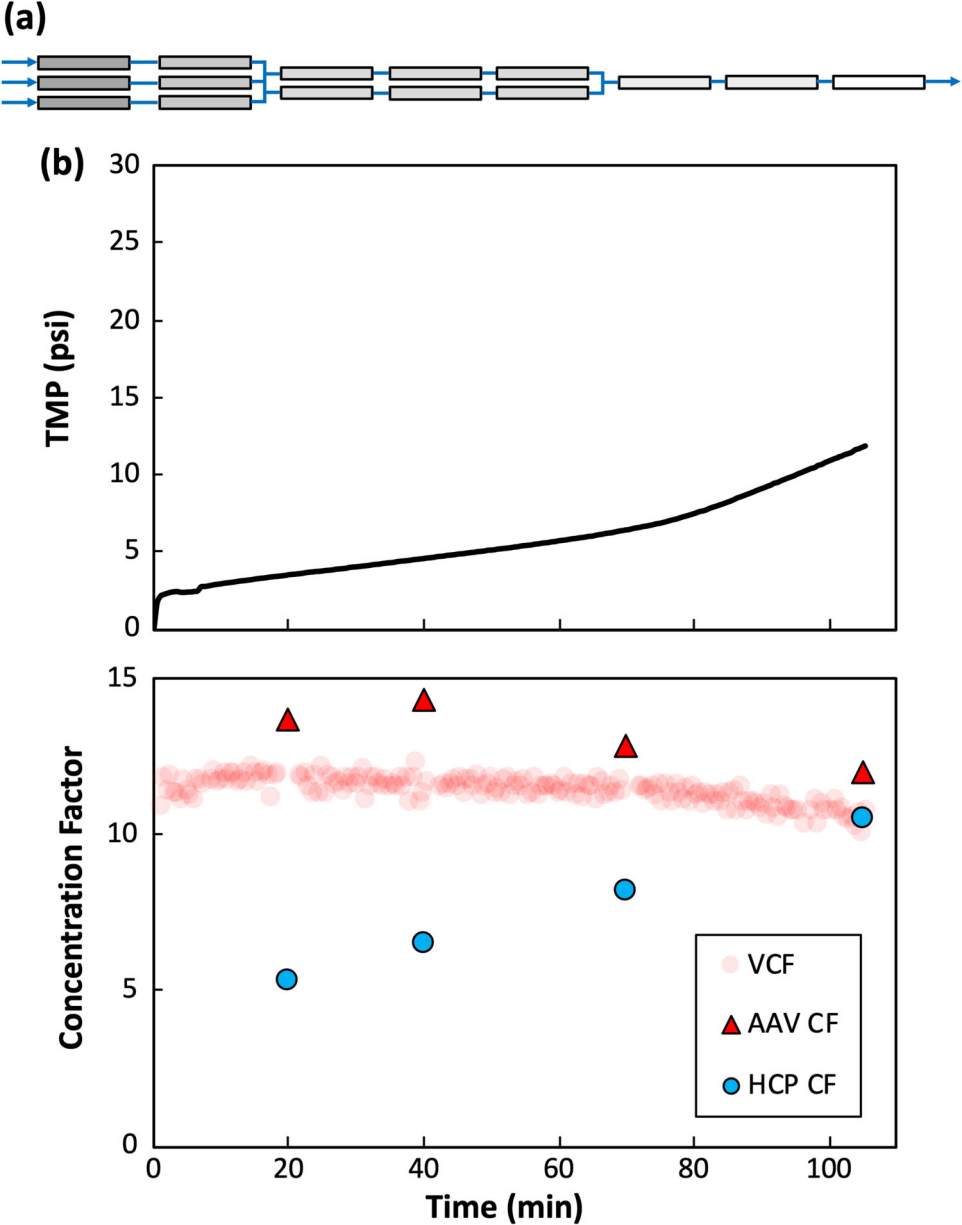
(a) Schematic of 8‐stage Centramate® SPTFF module with a 3‐3‐2‐2‐2‐1‐1‐1 configuration. (b) TMP (top panel) and volume, AAV, and HCP concentration factors (bottom panel) vs time for 8‐stage SPTFF using 100 kDa PES membranes.

Additional SPTFF experiments were then performed at pilot scale to demonstrate the robustness of SPTFF at larger process scale. For this study, AAV was harvested from HEK293 host cells that were lysed and clarified at Spark Therapeutics (Philadelphia, PA). 10 liters of AAV CCL was generated as feed material for SPTFF containing 1.79 × 10^11^ vg/mL AAV and 0.25 mg/mL HCP (DNA content was not quantified); the lower HCP content compared to the feed in the bench scale experiments likely reflects the difference in processing conditions and AAV serotype. The SPTFF module was built using fifteen Centramate® T02 cassettes with 100 kDa PES membranes (*Cytiva Life Sciences*), providing a total membrane area of 0.279 m^2^. The membranes were arranged across 8 serial stages in a 3‐3‐2‐2‐2‐1‐1‐1 “tree” configuration as shown in Figure 5a (Thakur and Rathore 2021). The reduction in membrane area towards the system outlet compensates for the reduction in volumetric flow rate as the CCL passes through the module. The SPTFF system was operated at a feed flow rate of 94.6 mL/min (feed flux of 20.3 LMH) and a retentate flow rate of 8.2 mL/min, which corresponds to a 12× VCF. A 143 mL buffer chase was performed at the end of the run to recover any AAV that accumulated within the boundary layer. The total retentate volume at the end of the run (after mixing the retentate with the buffer chase) was 1 liter, corresponding to a 10× overall volume reduction.

The TMP during SPTFF remained below 100 kPa (15 psi) over the entire run, which is well below the maximum recommended TMP of 60 psi for the Centramate® cassettes (top panel of Figure 5b). The TMP curve is concave‐up, with an increase in the rate of fouling occurring around 80 min into the experiment. The AAV concentration factor measured by qPCR (bottom panel of Figure 5b) is in good agreement with the VCF, indicating that there was minimal AAV accumulation within the system. The slightly larger value of the AAV concentration factor is likely an artifact associated with accuracy of the qPCR assay when measuring AAV titer in CCL due to media matrix interference. Overall, SPTFF was successfully operated with > 99% AAV yield (of which 4% AAV were recovered by the buffer chase). The HCPs are also concentrated by SPTFF, although the concentration factor is much smaller than that for the AAV due to HCP transmission through the membrane (corresponding to 37% HCP removal). The increase in the HCP concentration factor over the course of the experiment is likely due to membrane fouling that increases HCP retention.

### 3.6 Integrated Process Proof‐of‐Concept

Following the successful demonstration of SPTFF at pilot‐scale, an integrated process was designed to showcase the improvements in productivity that are gained by linking the clarification, SPTFF, and affinity chromatography steps together. Clarification was performed with two 0.027 m^2^ Clarisolve MS20 depth filters (*Millipore‐Sigma*) connected in parallel, with a 0.2 μm pore‐size Opticap XL 300 SHC (*Millipore‐Sigma*) filter connected inline for bioburden reduction. SPTFF was performed using the same 8‐stage system as described previously, with the retentate product then passed through a second 0.2 μm pore‐size Opticap XL 150 SHC filter (*Millipore‐Sigma*). Affinity chromatography was performed using AVB Sepharose High Performance (HP) resin (*Cytiva Life Sciences*) packed into a 24 mL column with 8 cm bed height.

7.8 liters of AAV lysate were fed to the integrated process at a flow rate of 135 mL/min. SPTFF was operated at a feed flow rate of 97 mL/min, with the retentate flow rate set to 8 mL/min to achieve a 12× inline VCF. A buffer flush of 140 mL was used to recover any AAV from the SPTFF module. The retentate was then purified by affinity chromatography using a loading flow rate of 6.6 mL/min. The integrated process was performed by staggering the start of the three unit operations to prevent drawing a vacuum at any point: the SPTFF was started slightly after clarification, and affinity loading was initiated slightly after the start of SPTFF. Surge bags were placed in between the unit operations to protect against any process disruptions and to account for differences in flow rates (required because of the limited availability of column and membrane sizes – this could easily be optimized upon scale‐up). The entire integrated process took approximately 4.5 hours to complete, compared to the 22 hours required without SPTFF due to the much shorter loading time for the affinity column after preconcentration by SPTFF.

**Figure 6:**
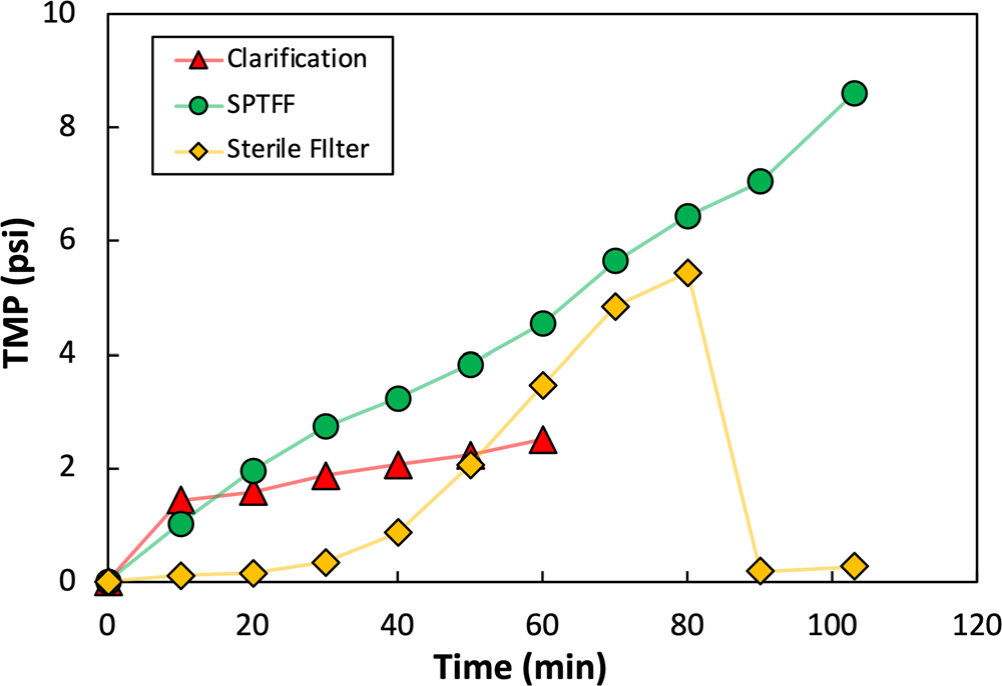
TMP measured during clarification, SPTFF, and bioburden reduction filter for the integrated run. Discontinuity in TMP for the Opticap XL 150 SHC is due to switching to a backup filter 80 min into the run. “0 min” corresponds to the start of each unit operation.

The pressure profiles in the clarification, SPTFF, and bioburden reduction filter during the integrated run are shown in Figure 6. The TMP during clarification was relatively stable, exhibiting a gradient of less than 1.5 psi/hr between 20 and 60 min. The TMP gradient during SPTFF was significantly greater, with the TMP approaching 9 psi at the end of the run. A crude estimate of the SPTFF capacity (taken as the maximum volumetric throughput that maintains a system TMP below 200 kPa = 30 psi) was obtained by extrapolating the TMP using a line of best fit between 70 and 100 minutes, giving a capacity of 125 L/m^2^. This suggests that as much as 34 L of material could have been processed through the integrated system at this scale. Fouling on the Opticap XL 150 SHC sterile filter (placed inline immediately after the SPTFF) was managed by switching to a backup filter 80 minutes into the run when the TMP reached ≈6 psi. This can be avoided entirely by increasing the sizing of the membrane area for the sterile filter in future runs.

The performance of the intensified process was compared to a parallel 500 L commercial scale process, which did not include SPTFF and relied on batch unit operations. The feed material for both processes came from the same bioreactor harvest to avoid batch‐to‐batch variability. Additionally, both processes maintained a similar flux through the depth filter clarification (167 ± 17 LMH) and the same residence time in the affinity column (~3 minutes). After implementing the SPTFF process to reduce the loading volume, the affinity column was loaded within just 5 h—equivalent to 25% of the allowable CCL hold time—and the resin utilization improved from 50% to 75%, while process yield increased significantly from 70% to approximately 100% based on inline samples. Although additional studies will be required to precisely define the maximum resin utilization that can be attained following the 12× preconcentration via SPTFF, these results provide initial evidence of enhanced resin utilization under intensified conditions while maintaining/improving product yield. Overall AAV yield through both processes was comparable. As presented in Table 4, the intensified process also provides an 81% reduction in overall processing time, primarily driven by the shorter loading time for the affinity column due to the 10X volume reduction provided by SPTFF. Overall resin productivity, defined as the mass of AAV ($M_{\mathrm{AAV}}$) processed per unit volume of resin ($V_{\mathrm{resin}}$) per unit time (t):

(3) $$\mathrm{Productivity}=\frac{M_{\mathrm{AAV}}}{V_{\mathrm{resin}}*t}$$

was 8.5x greater for the intensified process compared to the 500 L batch process.

**Table 4:** Performance comparison between 10 L intensified process and an equivalent 500 L commercial‐scale batch process.

Run	Clarification yield (%)	Affinity yield (%)	Overall yield (%)	Resin utilization (%)	Operation time (hr)	Productivity (vg/L_resin_/hr)
500 L Batch	69%	70 ± 7%	48 ± 5%	50 ± 5%	24	2.0 × 10^15^
Intensified	N/A	110 ± 10%**	51 ± 5%	75 ± 8%	4.5	1.7 × 10^16^

** Instantaneous yield reported based on an inline sample of material exiting the SPTFF step at steady state. Confidence intervals based on the average standard deviation associated with the AAV qPCR measurements.

### 3.7 Techno‐Economic Analysis

A simple techno‐economic analysis was conducted at 200 L harvest scale to compare the batch and intensified processes. Clarification used two parallel 0.55 m^2^ Clarisolve MS20 depth filters, with a 1.63 m^2^ Opticap SHC 0.2 μm bioburden reduction filter connected in series. The AAV titer exiting clarification was assumed to be 2 × 10^11^ vg/mL. The SPTFF step was designed to process 125 L/m^2^ of material at 10X VCF (post‐recovery) using 1.6 m^2^ of membrane area. Assuming a membrane cost of $6,400 per m^2^ of membrane area (as reported by McCarney et al. (2025) for 100 kDa PES flat sheet T‐series Omega cassettes), the 1.6 m^2^ of required membrane area would cost approximately $10,200 USD. Bioburden reduction following SPTFF at 200 L scale was designed using three Opticap XL 600 SHC filters in parallel to ensure manageable fouling – this contributed only an additional $600 to the intensified process cost.

**Figure 7:**
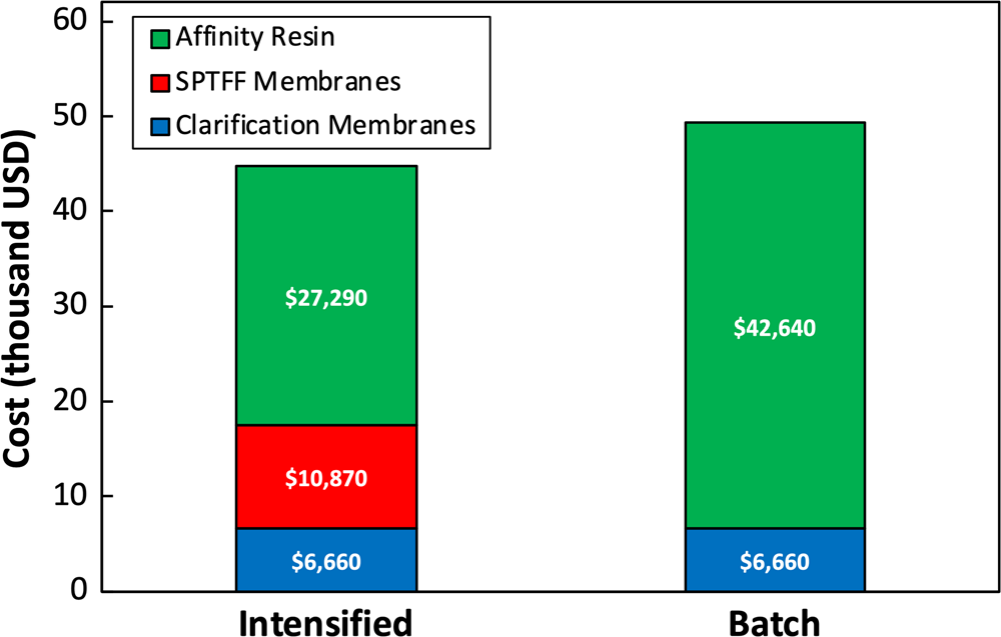
Estimated cost (in thousands of USD) for the depth filtration membranes, SPTFF membranes, affinity resin, and buffer components for intensified and batch AAV processes performed at 200 L scale. Economic calculations assume all raw materials are discarded after a single‐use.

Figure 7 summarizes the cost estimates of raw materials for the batch and intensified processes. Both processes employ the same clarification step at a cost of ≈$6700 USD. Raw materials costs associated with the affinity chromatography were calculated assuming the cost of AVB Sepharose High Performance (HP) resin is $30,700 USD per liter [Cytiva Website] with an AAV loading of 4.8 × 10^16^ vg/L_resin_ for the batch process and 7.5 × 10^16^ vg/L_resin_ for the intensified process based on the results in Table 4. The intensified process also provides a small reduction in buffer costs due to the smaller affinity column volume and the corresponding reduction in wash and elution volumes. This difference in buffer consumption is minimal ($1996 USD for the intensified process vs $2399 USD for the batch process) and was thus omitted from Figure 7. Although the intensified process requires the additional cost for the SPTFF, as well as the additional membrane area for the bioburden reduction step, these costs are more than offset by the reduced costs of the affinity resin. Overall, the intensified process offers approximately 9.1% lower cost of goods across these three unit operations compared to an equivalent batch process.

Based on prior internal data, we have found that labor contributes minimally to overall AAV process costs. We therefore did not explicitly include labor in the cost breakdown, although these costs would likely be reduced after SPTFF given the significantly shorter operating time of the intensified process (Table 4). Additionally, cost savings associated with improved facility utilization (e.g. reduced operational downtime) for the intensified process were not considered; the cost analysis shown in Table 4 assumes comparable labor and facility utilization for the batch and intensified processes. Lastly, all economic calculations assume that membranes and resins are discarded after a single use, with only items such as the chromatography skid and pumps reused. This means that costs associated with equipment depreciation would not differ significantly between the two processes.

## 4 Conclusions

This study provides the first examination of the use of single pass tangential flow filtration to intensify the downstream process for AAV manufacturing, focusing on the impact on the affinity chromatography step. Bench scale process development began by employing BioOptimal™ MF‐SL tangential flow microfilters to clarify 300 mL of AAV2 lysate with high AAV yield. The BioOptimal™ MF‐SL permeate was then further clarified using a 3M™ Polisher ST membrane adsorber, which offers a combination of high AAV yield along with high HCP and DNA removal given sufficient mass loading. The TFF‐based clarification process developed at bench scale may be used in perfusion applications, potentially intensifying the downstream process by enabling continuous upstream harvest.

The clarified lysate was then fed into a bench‐scale SPTFF system, which employed two Pellicon XL50 cassettes with 100 kDa Ultracel (regenerated cellulose) membranes. The low impurity content within the clarified lysate allowed SPTFF to be operated under conditions of negligible membrane fouling while achieving a VCF as high as 12×. Bench scale affinity chromatography was performed with both unconcentrated and preconcentrated material to showcase the productivity improvements that may be gained via SPTFF. AAV yield was high in both cases; however, the SPTFF‐preconcentrated material was processed in just 66 min and with a higher binding capacity, significantly improving the overall process economics.

Additional experiments were performed at pilot scale to evaluate the effectiveness of SPTFF to concentrate large volumes of AAV feed material. Multi‐staged SPTFF modules were able to concentrate 10 L of AAV CCL down to 1 L in a single pass while maintaining > 99% AAV yield and manageable membrane fouling. A pilot‐scale integrated process was designed by linking the clarification, SPTFF, and affinity chromatography steps. The integrated process displayed an 81% reduction in overall process time and 36% improved resin utilization. A simple techno‐economic analysis suggests that these productivity improvements should reduce the cost of goods by approximately 10% (without considering the difference in labor costs).

There are likely other opportunities to further optimize the SPTFF step in the intensified process. For example, the use of SPTFF membranes with larger pore‐size may allow for operation at higher fluxes and with greater impurity removal than the 100 kDa PES membranes used in our pilot‐scale runs (Chaubal, Yehl, et al. 2025). It is also likely that the SPTFF modules can be effectively cleaned and validated for multiple uses, which would significantly reduce the cost of that step (Bisschop 2019). Regardless, the increase in productivity associated with the incorporation of SPTFF in AAV processing has the potential to pave the way towards more affordable and accessible AAV gene therapies.

## Author Contributions

**Akshay S. Chaubal:** conceptualization, data curation, methodology, formal analysis, investigation, writing – original draft. **Ronny Horax:** data curation, methodology, formal analysis, investigation, writing – review and editing. **Christopher J. Yehl:** conceptualization, data curation, methodology, formal analysis, investigation, supervision, writing – review and editing. **S. Ranil Wickramasinghe:** conceptualization, funding acquisition, supervision, writing – review and editing. **Xianghong Qian:** conceptualization, funding acquisition, supervision, writing – review and editing. **Lu Wang:** supervision, resources, writing – review and editing. **Andrew L. Zydney:** conceptualization, data curation, methodology, supervision, writing – review and editing.

## Conflicts of Interest

The authors declare no conflict of interest.

## Supporting information


**Supplementary Figure S1:** TMP vs volumetric throughput during SPTFF with 148 mL of clarified AAV2 taken from the 25 cm^2^ 3M ™ Polisher ST capsule. SPTFF was operated with two cassettes in series at a feed flux of 6 LMH and an inline VCF of 11. **Table S1:** Performance results following an additional clarification run using BioOptimal™ MF‐SL filter. **Table S2:** Performance results following an additional SPTFF run at bench scale. SPTFF was operated with two Pellicon XL 100 kDa regenerated cellulose cassettes in series at a feed flux of 6 LMH. 120.41 mL of material was fed into the system, with 13.89 mL collected in the retentate and 14.46 mL of clean buffer used for recovery.

## Data Availability

The data that support the findings of this study are available from the corresponding author upon reasonable request.
