# Supplementary material for: Downstream Process Intensification for AAV Purification by Affinity Chromatography Using Single Pass Tangential Flow Filtration
Source: Biotechnol Bioeng. 2025 Oct 25;123(1):174–85. doi: 10.1002/bit.70090 (PMC12699131; doi:10.1002/bit.70090)
Supplement: Supplementary file 1 — Supplementary Figure S1: TMP vs volumetric throughput during SPTFF with 148 mL of clarified AAV2 taken from the 25 cm2 3M ™ Polisher ST capsule. SPTFF was operated with two cassettes in series at a feed flux of 6 LMH and an inline VCF of 11. Table S1: Performance results following an additional clarification run using BioOptimal™ MF‐SL filter. Table S2: Performance results following an additional SPTFF run at bench scale. SPTFF was operated with two Pellicon XL 100 kDa regenerated cellulose cassettes in series at a feed flux of 6 LMH. 120.41 mL of material was fed into the system, with 13.89 mL collected in the retentate and 14.46 mL of clean buffer used for recovery. [file BIT-123-174-s001.docx]

**Supplementary Information**

**Table S1** – Performance results following an additional clarification run using BioOptimal^TM^ MF-SL filter.

| **Fractions** | **Volume (mL)** | **Turbidity (NTU)** | **qPCR titer** | **HCP (mg/mL)** | **DNA (ug/mL)** |
| --- | --- | --- | --- | --- | --- |
|  |  |  | **(vg/mL)** |  |  |
| Feed | 349 | 552 | 6.57 ± 0.35 x 10^10^ | 1.36 ± 0.03 | 25.7 ± 2.3 |
| Permeate | 234 | 64 | 5.85 ± 0.22 x 10^10^ | 1.12 ± 0.05 | 18.2 ± 0.5 |
| Retentate | 115 | >>> | 8.59 ± 0.16 x 10^10^ | 1.59 ± 0.03 | 35.1 ± 2.1 |
| Buffer Chase Permeate | 24 | 39 | 4.68 ± 0.11 x 10^10^ | 0.23 ± 0.08 | 3.9 ± 0.1 |
| Buffer Chase Retentate | 17 | 268 | 2.14 ± 0.01 x 10^9^ | 0.13 ± 0.01 | 4.1 ± 0.4 |

**Supplementary Figure S1:** TMP vs volumetric throughput during SPTFF with 148 mL of clarified AAV2 taken from the 25 cm^2^ 3M^TM^ Polisher ST capsule. SPTFF was operated with two cassettes in series at a feed flux of 6 LMH and an inline VCF of 11.

**Table S2** – Performance results following an additional SPTFF run at bench scale. SPTFF was operated with two Pellicon XL 100 kDa regenerated cellulose cassettes in series at a feed flux of 6 LMH. 120.41 mL of material was fed into the system, with 13.89 mL collected in the retentate and 14.46 mL of clean buffer used for recovery.

|  | **Volume Reduction Factor** | **AAV Concentration Factor** | **AAV Yield** | **HCP Removal** |
| --- | --- | --- | --- | --- |
| **Before buffer chase** | 8.7 | 7.3 | 85% | 68 ± 5% |
| **After buffer chase** | 4.2 | 4.1 | 96% | 62 ± 6% |
